# Cardiac Autonomic Function Correlates with Arterial Stiffness in the Early Stage of Type 1 Diabetes

**DOI:** 10.1155/2011/957901

**Published:** 2011-07-24

**Authors:** S. Liatis, K. Alexiadou, A. Tsiakou, K. Makrilakis, N. Katsilambros, N. Tentolouris

**Affiliations:** First Department of Internal Medicine, Diabetes Center, Athens University Medical School and General Hospital of Athens “Laiko”, 11527 Athens, Greece

## Abstract

Arterial stiffness is increased in type 1 diabetes (T1D), before any clinical complications of the disease are evident. The aim of the present paper was to investigate the association between cardiac autonomic function and arterial stiffness in a cohort of young T1D patients, without history of hypertension and any evidence of macrovascular and/or renal disease. Large artery stiffness was assessed by measurement of carotid-femoral pulse wave velocity (PWV). Cardiac autonomic function was assessed by the cardiovascular tests proposed by Ewing and Clarke. Patients with a high cardiac autonomic neuropathy score (≥4) had significantly higher PWV than those with a low score (0-1). A negative, heart rate-independent, correlation between PWV and heart rate variation during respiration was observed (*r* = −0.533,  *P* < 0.001). In multivariable analysis, *E*/*I* index was the strongest correlate of PWV (**β**-coefficient = −0.326, *P* = 0.002). Cardiac parasympathetic function is a strong predictor of large arterial stiffness, in young T1D patients free of macrovascular and renal complications.

## 1. Introduction

Arterial stiffness increases as a result of the aging process and as a consequence of many disease states, including diabetes mellitus (DM) [1–6]. Aortic stiffness has independent predictive value for total and cardiovascular mortality, coronary morbidity and mortality, and fatal stroke in various populations at high risk for cardiovascular disease [4–6]. Vascular compliance of both the large and small arteries is reduced in patients with type 1 diabetes (T1D), at an early stage of the disease and before any clinical macrovascular or microvascular complications are evident, therefore increasing their risk for cardiovascular events [7–9]. The early presence of increased arterial stiffness in patients with T1D is believed to be a consequence of the blood vessels exposure to the chronic detrimental effects of hyperglycemia [10–12]. 

Diabetic autonomic neuropathy (DAN) is a serious, often overlooked, complication of DM. Cardiovascular autonomic neuropathy (CAN) is the most studied form of DAN and its prevalence varies dramatically between cohorts of diabetic patients, due to differences in the methodology used for its diagnosis and the lack of method standardization [13–15]. It has been reported that subclinical autonomic dysfunction may occur within two years of the diagnosis in patients with T1D [16]. Reduced cardiovascular autonomic function has been associated with increased total mortality and cardiovascular morbidity as well as mortality [17, 18]. 

Few previous studies have explored the hypothesis that early impairment of the cardiovascular structure and function and early abnormalities of the autonomic nervous system in patients with T1D might be interrelated, and have shown conflicting results [19–22]. Although these abnormalities could also be separate and unrelated (despite occurring in parallel time periods) consequences of the diabetic hyperglycemic environment, they might also be reversible.

In view of these considerations, the aim of the present study was to investigate whether there is an association between cardiac autonomic function and large artery compliance in a cohort of patients with T1D, without hypertension and/or any clinical evidence of macrovascular or renal disease.

## 2. Patients and Methods

Patients with T1D were consecutively selected from the outpatient clinic of our institution. Inclusion criteria required that patients were under 40 years of age, with an HbA1c < 8%, and had no history of arterial hypertension, macrovascular disease, or renal disease, including microalbuminuria. Patients receiving statins were excluded from the study. Since the performance of reliable cardiovascular autonomic tests requires the exclusion of potential confounding factors, such us concomitant illnesses and drug use (b-blockers, other antiarrhythmic drugs, antidepressants, antihistamines, and diuretics) those have been carefully ruled out [23]. In addition, patients were advised to refrain from certain potentially confounding lifestyle activities (exercise, smoking, and caffeine intake), and not to eat in the morning of the examination. All participants were given explanations about the purpose and the procedures of the study and then signed an informed consent. The study was approved by the ethics committee of the Laiko University Hospital.

Arterial stiffness was assessed noninvasively using the estimation of pulse wave velocity (PWV) which has emerged as the “gold-standard” for the measurement of regional arterial stiffness [24]. The theoretical basis of PWV is described by the Moens-Korteweg equation: PWV^2^ = E*h/2r*ρ*; where E is the slope of stress-strain relationship for a given vessel, Young's modulus; *ρ* is the density of fluid; h/2r is the wall thickness/diameter. PWV was calculated by measuring the time taken for the arterial pulse to propagate from the carotid to the femoral artery. The SphygmoCor system was used [25], which involves an arterial tonometer for recording pressure waveforms. Propagation time is measured from the foot of the carotid waveform to that of the femoral waveform using sequential recordings referenced to fixed points of the cardiac cycle (the R wave of the electrocardiogram).

Cardiac autonomic function was assessed using the battery of cardiovascular tests, proposed by Ewing et al. [26] and recommended by the American Diabetes Association's (ADA) consensus statement on standardized measures in diabetic [23]. Heart rate response during respiration (the deep breathing test) was assessed by calculating the ratio of the maximum and minimum heart rates during six cycles of paced deep breathing (*E*/*I* index). Heart rate response to standing (standing test) was calculated as the ratio of the longest R-R interval (found at about beat 30) to the shortest R-R interval (found at about beat 15) after standing up (30 : 15 ratio). Heart rate response to the Valsalva maneuver (Valsalva test) was assessed by calculating the ratio of the longest R-R interval after the maneuver to the shortest R-R interval during or shortly after the maneuver (VM index). All calculations were undertaken by measuring ECG recordings of RR intervals automatically, using the computer-aided examination and evaluation system VariaCardio TF4 (Medical Research Limited, Leeds, UK) [27]. 

The heart rate-based tests were evaluated according to published age-related tables [28]. Orthostatic hypotension was diagnosed when a fall in systolic blood pressure >20 mmHg was observed; a fall of 11–20 mmHg was considered as borderline and a fall of <10 mmHg as normal response. Each normal autonomic function test was graded as 0.0, each borderline test as 1.0 and each abnormal test as 2.0. On the basis of the sum of this score, the total CAN score was calculated as the sum of the partial scores (minimum: 0, maximum: 8). CAN was diagnosed when two out of the four tests performed were abnormal [23]. 

All tests were carried out between 07:00 and 09:00 h, in a quiet environment with stable temperature (22–24°C). All PWV and CAN measurements were assessed by the same person.

Statistical analysis was performed using the statistical package SPSS 15.0.1 (SPSS Inc., Chicago, IL, USA). All data were assessed for normal distribution of the values. Categorical data were compared using a Chi-square test. Comparisons of normally distributed data, between groups, were performed by the independent samples Student's *t*-test or by ANOVA. Simple correlations were performed using Pearson's or Spearman's correlation coefficient, as appropriate. Multivariable stepwise linear regression analysis was used to assess the independent contribution of variables possibly associated with PWV. *P* values (two-tailed) < 0.05 were considered statistically significant.

## 3. Results and Discussion

A total of 66 patients were included in the study, out of whom 31 (46.97%) were men. The mean age was 27.1 years while the mean duration of diabetes was 12.3 years. All patients were under intensive insulin treatment (basal-bolus regimens) and 11 (16.66%) received continuous subcutaneous insulin infusion via an insulin pump. The patients had decent glycemic control (mean HbA1c: 7.4%). The demographic and clinical characteristics of the study participants are shown in detail in Table 1. Seven patients fulfilled the criteria of CAN diagnosis (score ≥ 4). Only four patients had an abnormal Valsalva test while 17 had an abnormal deep breathing test. Data for Valsalva index are missing for five patients due to poor compliance in performing the test. The mean values of PWV and the indices of cardiac autonomic function are shown in Table 2.

Patients with a high total CAN score (≥4) had a significantly higher PWV value than those with a low total score (0-1; Table 3). PWV was significantly higher in patients with abnormal deep breathing and standing-up tests, compared to patients with normal result tests while there was no such difference observed regarding the Valsalva maneuver test and the orthostatic blood pressure test (Table 3). After adjustment for age, gender and duration of diabetes, the difference in PWV between patients with normal and abnormal tests remained significant only for the deep breathing test (*P* = 0.012) while borderline significance was observed regarding patients with high versus low total CAN score (*P* = 0.067).

In univariate analysis, PWV correlated positively with age (*r* = 0.527, *P* < 0.001), diabetes duration (*r* = 0.378, *P* = 0.002), systolic blood pressure (*r* = 0.278, *P* = 0.024), diastolic blood pressure (*r* = 0.335, *P* = 0.006), waist circumference (*r* = 0.293, *P* = 0.017), and waist-to-hip ratio (*r* = 0.378, *P* = 0.001). No significant association was found between PWV and gender, BMI, smoking status, HbA1c, and plasma levels of HDL-C, LDL-C, and triglycerides.

**Figure 1:**
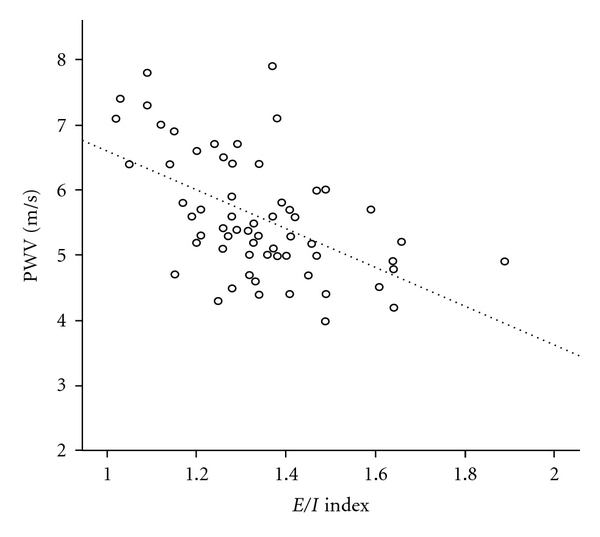
Simple linear correlation between pulse wave velocity and *E*/*I* index.

A significant negative correlation was observed between PWV and the *E*/*I* index (*r* = −0.533, *P* < 0.001; Figure 1) that was modestly attenuated when adjusting for heart rate (*r* = −0.452, *P* < 0.001). A weaker negative correlation was observed between PWV and the 30 : 15 index (*r* = −0.275, *P* = 0.025), which was vanished after adjusting for heart rate (*r* = −0.137, *P* = 0.29). No significant correlation was observed between PWV and the Valsalva index as well as the change in blood pressure after standing up. After adjustment for age, PWV remained significantly correlated only to diastolic blood pressure (*r* = 0.296, *P* = 0.017), waist-to-hip ratio (*r* = 0.293, *P* = 0.018), and *E*/*I* index (*r* = 0.401, *P* = 0.001).

In multivariable linear regression analysis, with PWV as dependent variable, a multivariable model including the above significantly correlated to PWV parameters was created (Table 4). In this model, age, waist-to-hip ratio, and *E*/*I* index were independently associated with PWV. *E*/*I* index was the strongest predictor of PWV (standardized *β*-regression coefficient = −0.326, *P* = 0.015). *E*/*I* index, age and waist-to-hip ratio accounted for 43.7% of PWV variability (Table 4).

### 3.1. Discussion

The main finding of the present study is that the compliance of large arteries assessed by measurement of PWV in patients with T1D, even at a relatively early stage of the disease, is related to certain indices of parasympathetic autonomic nervous system function, principally the beat-to-beat variation in heart rate with respiration.

The participants of the present study were carefully selected in order to avoid the potential effect of diabetic complications, both on PWV and autonomic function. Hence, participants were young (<40 years of age), had no history of hypertension, and were free of macrovascular complications and nephropathy, including microalbuminuria. In addition, all patients were treated with intensive insulin therapy (either multiple daily injections or continuous subcutaneous insulin infusion), having a fairly decent glycemic control (mean HbA1c: 7.4%). It is known that renal disease, even in the microalbuminuric stage, is related to elevation of blood pressure, decreased blood pressure decline during sleep, increased arterial stiffness, and impaired autonomic nervous system function [29–31]. Hence, the exclusion of patients with any degree of diabetic nephropathy was important in order to eliminate any potential confounding effect of nephropathy on either arterial compliance or autonomic function. 

According to our results, PWV correlated negatively to the *E*/*I* index (*r* = −0.533) and (to a lower extent) the 30 : 15 ratio (*r* = −0.275) but not to the VM index (*r* = −0.119). After adjustment for heart rate at rest, the association of *E*/*I* index and PWV remained significant (*r* = −0.452, *P* < 0.001), while the 30 : 15 ratio was not related significantly to PWV. After full adjustment for possible confounding factors, the association between *E*/*I* index and PWV not only remained significant (Table 4), but *E*/*I* index was the strongest predictor of PWV in the model.

The deep breathing test (expressed by the *E*/*I* index) is considered as the most reproducible of the cardiac autonomic function tests [32]. Interestingly, further analysis of the data showed that the VM index was affected by smoking status, being significantly lower in smokers. In addition, smoking status affected the relation between VM index and PWV, being of borderline significance in nonsmokers (*r* = −0.288, *P* = 0.07) but neutral in smokers. It has been previously reported that smoking may attenuate the Valsalva heart rate response, due to dysfunction in some part of the autonomic reflex arch [33].

Both the deep breathing test and the standing-up test (as it concerns heart rate variation), reflect mainly parasympathetic activity of the heart innervation (although the sympathetic nervous system may partly affect these measures) [34, 35]. On the other hand, heart rate variation during the Valsalva maneuver is mediated through alternating activation of parasympathetic and sympathetic nerve fibres [35]. 

Previous cross-sectional studies have demonstrated an association between arterial stiffness and autonomic neuropathy in both types of diabetes (type 1 and type 2) [19–22, 36]. In the study by Ahlgren et al., a significant correlation between *E*/*I* index and aortic distensibility (measured noninvasively by ultrasound) was found in females (but not in males) with type 1 diabetes [20]. Patients with established nephropathy (both macro- and microalbuminuria) were included. In the present study, the association between PWV and autonomic function indices was gender independent. It has to be noted, however, that due to the small population size, we were not able to perform gender-specific analysis, and a potential gender effect might have been missed. 

 In a subsequent Dutch study by van Ittersum et al. [22], performed in a cohort very similar to our population (patients with T1D without microalbuminuria), it was found that large artery compliance was related to the shortening of the RR interval of the ECG after standing up, but not to the heart rate variation during respiration. On the contrary, in our cohort, a strong negative correlation of large arterial stiffness to the heart rate variation during respiration was observed. On the other hand, we also found a (negative) correlation of PWV to the RR shortening after standing up, but this was weak and disappeared after adjustment for resting heart rate. Some differences between the two studies might explain this discrepancy: first (1), in the Dutch study, heart rate variation during respiration was expressed as the maximal *difference* in duration of the RR interval of the ECG during deep inspiration and expiration while in the present study the same parameter was expressed as the *ratio* between the two respective values; second (2), Van Ittersum et al. performed adjustment for 24-hour heart rate while we only had one measurement of resting heart rate available; third (3), the demographic and clinical characteristics of patients included in the present study were suggestive of an even “earlier” T1D population than in the study by van Ittersum et al. Indeed, our cohort was younger (27.1 ± 6 versus 32.6 ± 11 years) and had a shorter duration of diabetes (12.3 ± 7.7 versus 14.6 ± 10 years). In addition, patients in our study had better glucose control than in the Dutch study (HbA1c: 7.4 ± 4.5 versus 8.1 ± 1.4). As a consequence, the mean PWV values of the two populations was different, with a (close to normal) 5.6 ± 0.9 m/s in our study, compared to a clearly higher value of 6.5 ± 1.8 m/s in the study by van Ittersum et al. It can be assumed that the presence of a less advanced disease in our population may have allowed the association between parasympathetic activity (expressed as the *E*/*I* ratio) and arterial stiffness to emerge.

The association between PWV and cardiac autonomic function has been previously examined in a small study in healthy individuals as well [37]. Similarly to our results, moderate correlations were found between autonomic indices expressing mainly parasympathetic activity and PWV. Healthy volunteers participating in that study, were older than our cohort (mean age 38.2 ± 11.2 years). 

Both the decrease of large arteries elasticity and the dysfunction of autonomic nervous system appear early in the natural history of T1D [7–9]. The question arising from the present (and the previously mentioned) study is which of the events (autonomic dysfunction and stiffness of the large arteries) appear first and whether there is a causative relationship between each other. Two reasonable hypotheses may be generated: arterial stiffness may produce dysfunction of the cardiac autonomic nervous system or (the other way round) CAN may induce stiffness of the large arteries. Of course, a noncausal relation may also be the case, since the two conditions may develop in parallel as consequences of ageing and accelerated because of the “toxic” hyperglycemic environment. In the present study, HbA1c was not associated with PWV (*r* = 0.072, *P* = 0.59). It might be hypothesized, however, that a single measurement of HbA1c, undertaken some years after the initiation of diabetes, might not reflect the overall glycemic burden, acquired during the entire course of the disease.

A possible mechanism that might explain the first hypothesis is the impairment of baroreceptor function, induced by stiffening of the arterial wall [38]. On the other hand, dysfunction of the cardiovascular autonomic system may influence the elasticity of the arterial wall by affecting the vascular tone of large arteries. In animal studies, it has been shown that the integrity of the autonomic nervous system is important in the preservation of the elastic properties of the aorta [39–42]. Most of the animal studies, however, implicated an acute modulation of the autonomic nervous system whereas, in patients with T1D, a chronic, gradual, and cumulative effect is present. Another mechanism possibly implicated in the induction of arterial stiffness by autonomic dysfunction is the increase in heart rate. Indeed, increases in heart rate per se result in stiffening of arteries independently of changes in activity of the autonomic nervous system [39, 43]. In the present analysis, the correlation between the *E*/*I* index and PWV remained highly significant after adjustment for heart rate, a finding indicating that the association between parasympathetic dysfunction and arterial stiffness is not mediated by an increase in heart rate. Finally, alterations in autonomic tone may lead to artery stiffening through trophic influence, leading to changing the structure of vessels [44, 45]. 

In the Pittsburgh Epidemiology of Diabetes Complications (EDC) study, it has been shown prospectively that CAN (expressed as the *E*/*I* ratio) is associated with increased arterial stiffness measures, 18 years later in life of patients with childhood-diagnosed T1D [46]. The authors of that study concluded that CAN may exert a major pathophysiological role in the development of arterial stiffening in patients with T1D. The main limitation of the present study is its cross-sectional design and the small population size. However, the latter, is partly overcome by the homogeneous characteristics of the patients, being of a young age, relatively short disease duration, decent glycemic control, and, most importantly, free of macrovascular and renal complications of diabetes.

## 4. Conclusions

According to the results of the present study, cardiac autonomic function, particularly parasympathetic activity of the heart, as expressed by heart rate variation during respiration, correlates strongly to large arterial stiffness in patients with type 1 diabetes. Most interestingly, this finding was observed in a population of young patients, without history of hypertension, with relatively short disease duration and free of macrovascular and/or renal complications. A prospective followup of these patients might provide further information regarding the progression of both cardiac autonomic function (including the development of cardiac autonomic neuropathy) and arterial stiffness.

## Figures and Tables

**Table 1:** Demographic and clinical characteristics of the study population.

	*N* (%)/mean (SD)
Gender (males/females)	31 (47)/35 (53)
Age (years)	27.1 (6.0)
BMI (kg/m²)	24.2 (3.1)
Diabetes duration (years)	12.3 (7.7)
Current smoking	23 (35)
HbA1c (%)	7.4 (4.5)
Type of insulin treatment (MDI/CSII)	55 (83.3)/11 (16.7)
SBP (mmHg)	120.9 (13.1)
DBP (mmHg)	78.5 (12.5)
LDL-C (mg/dL)	99.8 (23.5)
HDL-C (mg/dL)	60.8 (13.4)
Triglycerides (mg/dL)	63.2 (32.1)

SBP: systolic blood pressure, DBP: diastolic blood pressure, MDI: multiple daily injections, CSII: continuous, subcutaneous insulin infusion.

**Table 2:** Pulse wave velocity and indices of autonomic function in the study population.

	*N* (%)/mean (SD)
Pulse wave velocity (m/s)	5.6 (0.9)
*E*/*I* index	1.33 (0.16)
*E*/*I* index (normal/borderline/low)	39/10/17 (59.1/15.2/25.7)
30 : 15 index	1.33 (0.18)
30 : 15 index (normal/borderline/low)	51/5/10 (77.3/7.6/15.1)
Valsalva index	1.94 (0.40)
Valsalva index* (normal/borderline/low)	50/7/4 (75.8/10.6/6.1)
Change in SBP (mmhg)	−0.36 (9.84)
Change in SBP (normal/borderline/low)	58/5/3 (87.8/7.6/4.6)
Total score (median)	1.0 (0.0–7.0)
Presence of CAN (yes/no)	7/59 (10.6/89.4)

*Not available in 5 patients due to poor compliance in performing the test.

**Table 3:** Mean (SD) PWV value in patients with normal, borderline, and abnormal autonomic nervous function tests. Total score is considered normal when ≤1, borderline when 2-3, and abnormal when ≥4.

		Normal result	Borderline result	Abnormal result	*P* for trend
PWV (m/sec)	Deep breathing test (*E*/*I* index)	5.37	5.53	6.19**	0.006
	Orthostatic test (30 : 15 ratio)	5.46	6.38*	5.96	0.038
	Valsalva test (VM index)	5.52	5.36	5.48	0.89
	Orthostatic test (BP change)	5.55	5.68	6.43	0.27

	Total score	5.47	5.58	6.35*	0.042

*P < 0.05, **P < 0.01, for comparisons of PWV values between abnormal/borderline test and normal test (adjusted for multiple comparisons using Bonferroni adjustments).

**Table 4:** Multivariable linear regression model with pulse wave velocity set as dependent variable. Additional variables included in the model (and turned to be nonsignificant) were gender, duration of diabetes, and diastolic blood pressure.

Variable	*β*-coefficient	Standardized *β*-coefficient	*P*	95% CI (for *β*)
Age	0.048	0.317	0.005	0.016, 0.081
Waist-hip ratio	2.625	0.218	0.033	0.216, 5.033
*E*/*I* index	−1.930	−0.326	0.002	−3.110, −0.750

*R* ^2^ = 0.437.
